# Tissue Sodium Accumulation: Pathophysiology and Clinical Implications

**DOI:** 10.3390/antiox11040750

**Published:** 2022-04-09

**Authors:** Endre Sulyok, Bálint Farkas, Bernadett Nagy, Ákos Várnagy, Kálmán Kovács, József Bódis

**Affiliations:** 1 National Laboratory for Human Reproduction, University of Pécs, 7624 Pécs, Hungary; esulyok@t-online.hu (E.S.); nagy.bernadett@pte.hu (B.N.); varnagy.akos@pte.hu (Á.V.); kovacs.kalman@pte.hu (K.K.); bodis.jozsef@pte.hu (J.B.); 2 ELKH-PTE Human Reproduction Scientific Research Group, 7624 Pécs, Hungary; 3 Faculty of Health Sciences, Doctoral School of Health Sciences, University of Pecs, 7621 Pécs, Hungary; 4 Department of Obstetrics and Gynecology, School of Medicine, University of Pecs, 7624 Pécs, Hungary

**Keywords:** salt intake, nonrenal regulation, tissue damage, oxidative stress, perinatal pathologies

## Abstract

Excessive sodium intake has been well established as a risk factor for the development and progression of cardiovascular and renal diseases. Its adverse effects are achieved by renal sodium retention and related volume expansion and by inducing low-grade inflammation and oxidative stress (OS) in the target tissues. This review presents the recent concept of nonosmotic sodium storage in the skin interstitium, the subsequent dissociation of sodium and volume homeostasis, and the cellular response to the increased tissue sodium concentration. Furthermore, data are shown on the sodium barrier and buffering potential of the endothelial glycocalyx that may protect the functional integrity of the endothelium when it is challenged by an increased sodium load. Finally, examples will be given of the involvement of oxygen free radicals (OFR) in sodium-induced tissue damage, and some clinical entities will be mentioned that are causally associated with sodium/volume retention and OS.

## 1. Introduction

**Figure 1:**
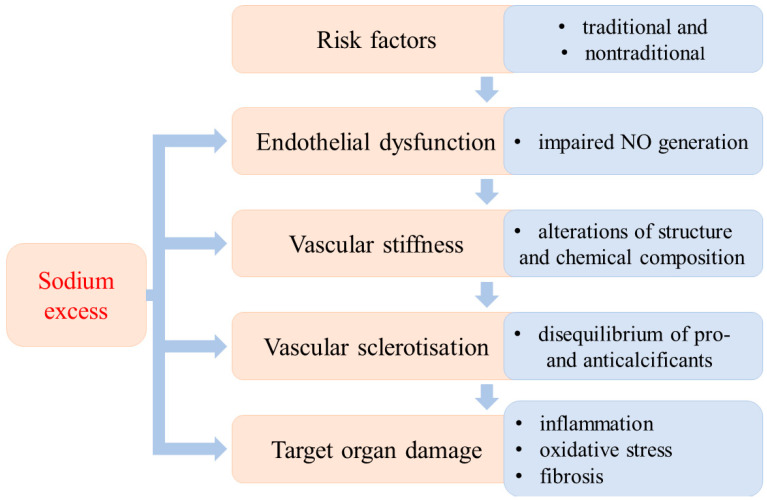
Sequential stages of the evolution of hypertensive disease and the impact of excess sodium.

The development of hypertension is a long-lasting process that is the result of the interactions of several well-defined factors. Different stages of its development have been established according to the dominant etiological factors. The traditional (obesity, dyslipidaemia, diabetes mellitus, inactivity, stress, increased activity of sympathetic nervous system, high salt intake, smoking) and nontraditional risk factors (i.e., low-grade inflammation, aggressive free radicals (AFR), glycation end products, vitamin D3 deficiency, asymmetric dimethylarginine (ADMA), low birthweight) may develop into hypertension. From a theoretical point of view, different stages of the evolution of hypertension can be established according to the dominant pathological alterations. Endothelial dysfunction is the first step, followed by vascular stiffness due to structural and chemical changes in the vascular wall. Vascular stiffness progresses to sclerotization and finally to target organ damage (Figure 1).

It is important to emphasize that these developmental stages cannot be clearly separated from each other; they do not always occur successively. In this regard, it is important to stress that the high dietary sodium intake can initiate or aggravate pathological processes in each stage of the evolution of hypertension [1,2,3].

Epidemiological, clinical, and experimental studies have demonstrated close positive correlations between salt intake and hypertension [4,5,6,7], and the reduction of salt intake resulted in a decrease in blood pressure in both hypertensive and normotensive subjects [8,9]. The low capacity of renal sodium handling has been proved to be the major determinant of the development and progression of salt-sensitive, volume-dependent hypertension [10].

Convincing evidence has been provided for the role of increased activity of the sympathetic nervous system in the development of cardiovascular diseases [11,12,13]. It has also been shown that salt sensitivity can be attributed to sympathetic overactivity, increased adrenaline release, and enhanced vascular sensitivity to α-adrenoreceptor activation rather than to reduced renal sodium excretion. In support of this contention, a ganglionic blockade with hexamethonium reduced blood pressure, but its reduction was more pronounced in salt-sensitive mice kept on a high-sodium diet for 21 days than in those on high sodium for a shorter period or in those on a normal salt diet [14]. Furthermore, a high-salt diet proved to potentiate the central actions of angiotensin II to induce sympathetic nervous outflow and to maintain hypertension [15].

Diabetes mellitus type 2 is of particular interest because it is associated with renal sodium retention. The mechanisms of enhanced renal sodium reabsorption have not been clearly established, but evidence has been provided for the involvement of epithelial sodium channel (ENaC)-mediated sodium reabsorption. This transport appears to be stimulated by the additive effects of aldosterone and the combined actions of hyperinsulinaemia and hyperglycaemia [16]. In addition to hyperinsulinaemia-mediated renal tubular sodium transports, it has also been suggested that the increased glomerular filtration of glucose may enhance the activity of the proximal tubular Na+-glucose co-transporter and may contribute to sodium retention [17].

Insulin has also been shown to play an opposing role in the control of endothelial function [18,19]. On one hand, it is vasoprotective as it stimulates NO generation by activating the phosphoinositide 3-kinase (PI3-K)-AKT-eNOS pathway. On the other hand, it activates the mitogen-activated protein kinase (MAPK)-dependent signaling pathway that regulates the secretion of vasoconstrictor endothelin-1 (ET-1). Under physiological conditions, these opposing endothelial effects of insulin are in balance while in pathologies associated with insulin resistance, insulin signaling is directed toward the MAPK-ET-1 pathway and may lead to endothelial dysfunction and may progress to a remodeling of the vascular wall and atherosclerotic lesions [20,21]. In the last decades, convincing evidence has emerged indicating the essential role of nonrenal mechanisms in the regulation of sodium balance [22].

In this review, we demonstrate the role of nonosmotic tissue sodium storage in blood pressure regulation and make an attempt to outline the control of sodium balance by the skin interstitium, endothelial glycocalyx, and placenta to demonstrate the possible involvement of excess sodium-induced OS in perinatal pathologies, including preeclampsia, perinatal programming of adult diseases, and bronchopulmonary dysplasia of preterm neonates. The impact of sodium accumulation has been analyzed in selected organs (kidney, heart, placenta) because sodium-induced tissue damage has been most extensively studied in these organs, and substantial evidence has been provided for their apparent clinical implications.

## 2. Nonosmotic Tissue Sodium Storage

### 2.1. The Function of Skin Interstitium

It has been generally accepted that the inefficient renal sodium excretion and the subsequent expansion of the extracellular space are the major factors in blood pressure elevation. This view, however, has been recently challenged because there have been reports of the dissociation of sodium and volume homeostasis. Sodium retention, due to high dietary NaCl intake, may not cause extracellular volume expansion in human subjects [23], and salt-induced hypertension may develop in salt-sensitive rats without body fluid expansion [24]. To explore the underlying mechanism(s) of these observations, Titze and his group developed the concept of reversible, nonosmotic sodium storage at tissue levels.

The dynamic conversion of osmotically active/inactive sodium serves as a buffer system to protect the volume of extracellular space and blood pressure. The major reservoir for the inactive sodium is the subcutaneous interstitium, but the bones, cartilages, and skeletal muscles have been shown to have a similar function. Glycosaminoglycans (GAGs), the negatively charged, large biopolymers, have been identified as the basis of sodium binding and storage. The binding capacity of GAGs depends on their amount, the degree of their polymerisation, and the charge density [25].

**Figure 2:**
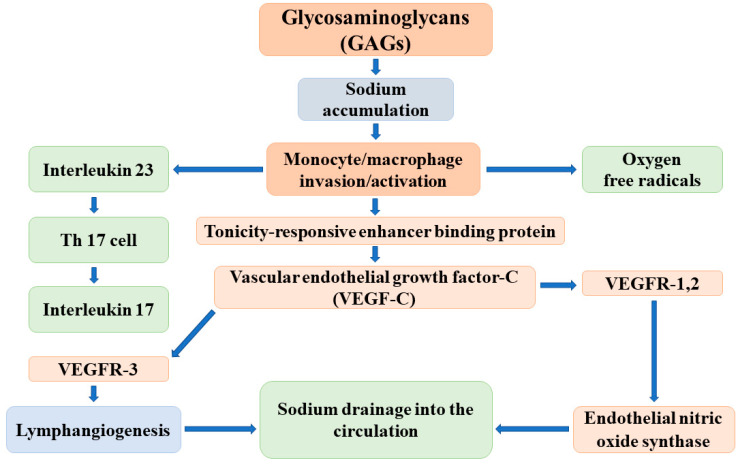
The possible mechanisms of the reversible, nonosmotic sodium storage and release in the skin interstitium.

The major elements of the regulatory cascade of reversible sodium storage in the skin interstitium have been identified by Titze and colleagues as follows: the negatively charged GAGs bind sodium and create hypertonicity in the immediate surroundings. The increase of tissue tonicity induces monocyte/macrophage invasion and activation. These activated cells express a tonicity-responsive enhancer binding protein (TonEBP) that stimulates vascular endothelial growth factor-C (VEGF-C) secretion. VEGF-C, via receptor VEGFR-3, increases lymphangiogenesis and the capacity of the lymphatic system to accommodate, as well as drain back into the circulation, the sodium and volume that are liberated and released into the interstitium. In addition, VEGF-C enhances the expression and activity of endothelial nitric oxide synthase (eNOS), which leads to the reduction of vascular resistance and blood pressure (Figure 2) [26].

The effective operation of this buffer system has been supported by animal experiments and clinical observations. In salt-sensitive Dahl rats, the capacity of the interstitium to bind osmotically inactive sodium is lower than that in controls; therefore, its buffer function is reduced, and excessive sodium intake results in volume expansion and in the development/worsening of hypertension [27]. Given these observations, the deletion of the TonEBP gene in mononuclear phagocytes decreases the mRNA and protein expression of VEGF-C and leads to the reduction of the lymphatic capillary network, the diminishment of interstitial chloride clearance, and the development of salt-sensitive hypertension [28].

Similar sodium buffer mechanisms have been assumed to operate in humans. Indeed, manipulations of salt intake in healthy subjects or in patients with chronic kidney diseases were associated with changes in VEGF-C levels corresponding to sodium intake [29]. Furthermore, the interruption of the VEGF-C signaling pathway with sunitinib/lucitanib in oncologic patients increased blood pressure [30]. This finding has been confirmed in animal models by demonstrating that angiogenesis inhibition induced interstitial salt accumulation and salt-sensitive hypertension [31].

The concept of the sodium buffer function in the skin interstitium is well established, attractive, and greatly appreciated. However, several questions remain to be addressed as follows: (a) negatively charged chloride is also stored in the skin interstitium in an amount equivalent to sodium [32] and, most likely, released simultaneously. The biochemical basis and the mechanisms of reversible Cl-storage remained unexplored. (b) A substantial fraction of dipole water molecules in the skin interstitium is motionally constrained by electrically charged macromolecules. By using H’-NMR freely moving, loosely and tightly bound tissue, water fractions can be differentiated (physical water compartments) [33]. It is reasonable to assume that when the amount, structure, and charge density of macromolecules change not only the sodium, but also the water, the binding changes. Therefore, not the water-free sodium alone but the sodium and water binding/release together can meet the actual need of volume regulation. (c) Sodium-induced monocyte/macrophage activation results in not only TonEBP and VEGF-C expression but also in the release of reactive oxygen species (ROS) and inflammatory cytokines that may limit NO production/availability [26]. (d) The regulation of building up and breaking down the interstitial macromolecules as well as the underlying mechanisms of reversible sodium binding and storage have not been revealed. It is most likely that, analogous to the renomedullary interstitium, these complex processes are controlled by vasoactive hormones involved in the classical volume- and osmoregulation [34,35,36,37].

Besides these unanswered questions, it is of great concern that the whole concept of nonosmotic sodium storage in the skin interstitium has been most recently challenged by Rossitto and coworkers. These authors presented convincing evidence that the tissue sodium excess is not hypertonic; therefore, biomedical stress, rather than osmotic stress due to oedema accumulation, is responsible for the activation of the lymphogenetic signal cascade [38]. Taking into account all these considerations, the paradigm shift from the classical two-compartment model to the recently developed three-compartment model of sodium and volume homeostasis needs to be further substantiated [39].

### 2.2. Endothelial Glycocalyx

Experimental and clinical studies have demonstrated that glycocalyx covering the luminal surface of the vascular endothelium also plays an essential role in the regulation of sodium homeostasis. It is a mesh-like structure that consists of glycoproteins, proteoglycans, and GAGs. Its volume and composition are dynamically regulated by the endothelial cells under the influence of circulating plasma. It functions to maintain vascular permeability and haemostasis, to protect the vascular wall by its antiatherogenic and anti-inflammatory properties, and to mediate flow-dependent NO synthesis. In addition, its negatively charged macromolecules have the capacity to bind and reversibly store sodium, providing a sodium buffer and first-line barrier against sodium overload of endothelial cells [40].

When the volume of the endothelial glycocalyx is reduced or its integrity is impaired, its capacity to bind and buffer sodium diminishes and the risk for the development of hypertension markedly increases. Several clinical conditions are known to damage the endothelial glycocalyx, but with respect to sodium homeostasis, the acute sodium/volume loading appears to be the most relevant [41]. Namely, in response to salt loading, the barrier function of the glycocalyx diminishes and more sodium reaches the luminal surface of endothelial cells, where it induces and activates epithelial sodium channels. As a result, sodium uptake by the cells increases, the cortex of the cells stiffens, and the endothelial NO generation decreases with the subsequent elevation of vascular tone [42]. As a mechanotransductor, the glycocalyx mediates flow-dependent vasorelaxation by stretching the glycocalyxlipid bilayer cytoskeleton system and increases NO production though the activation of TRP channels [43]. Further evidence for the vasculo-protective role of the glycocalyx is substantiated by the observations that its enzymatic elimination impaired but its supplementation with sulodexide improved vascular integrity and reduced the risk of the development of salt-sensitive hypertension [44,45].

As the cellular accumulation of sodium has a central role in the decreased NO production, it is relevant to postulate that sodium-induced OS may contribute to endothelial dysfunction and the initiation and progression of salt-sensitive hypertension.

In conclusion, the endothelial glycocalyx functions as a sodium buffer and first-line barrier to protect endothelial cells against increased sodium influx when exposed to excess circulating sodium. In clinical conditions characterized by a decreased sodium binding capacity of the glycocalyx, more sodium enters into the cells, causing impaired NO generation, elevated vascular resistance, and hypertension. Moreover, the sodium load would increase unbound, osmotically active sodium, resulting in water retention, volume expansion, and a further rise in blood pressure.

## 3. Sodium Loading and Oxidative Tissue Damage

### 3.1. Kidney

In a most recent study, the relationship between salt loading and the generation of reactive oxygen species (ROS) and the involvement of a transcriptional factor (Nuclear factor E2-related factor, Nrf2) were evaluated in the renal cortex and medulla tissue lysates. It has been found that high salt intake downregulated Nrf2 expression and the mRNA level of genes of antioxidative enzymes related to Nrf2. At the same time, salt loading significantly increased ROS levels. This observation provides a possible mechanism of sodium-dependent OS in renal tissue that may contribute to the deterioration of kidney function when challenged by salt load [46].

The importance of high salt intake in inducing OS has been further emphasized in rats by showing an increased excretion of 8-iso-prostane and malonyldialdehyde. Furthermore, increased renal cortical mRNA expression for elements (gp91phox and p47phox) of pro-oxidant nicotinamide adenine dinucleotide phosphate (NAD(p)H) oxidase and the reduced mRNA expression of antioxidant enzymes, superoxide dismutases (CuZnSOD and MnSOD), were demonstrated. For these studies, Sprague–Dawley rats kept on 6 g/kg NaCl [47,48] and Wistar rats fed with a 3% sodium diet for two weeks were used [49]. These findings strongly support the concept that the salt-induced ROS generation plays a major role in kidney damage and progressive decline in renal function.

Basic studies have revealed that elevated tissue sodium concentration activates immune reactions and accelerates autoimmune processes. Namely, it induces murine and human Th17 cells and activates the p38 mitogen-activated protein kinase (p38/MAPK) pathways that involve TonEBP and serum/glucocorticoid-regulated kinase (SGK1). Chemical inhibition or gene silencing of p38/MAPK pathways eliminate high salt-induced Th17 cell development and the subsequent upregulation of proinflammatory cytokines [50].

With these observations in line, Zhang et al. demonstrated that high salt intake increased the proinflammatory molecules but decreased anti-inflammatory and proendocytic molecules in human and mouse macrophages. That is, the expression of proinflammatory cytokines (IL β1, IL 8), chemokine ligands (CCL2, CCL8, CXCL1/2), and Toll-like receptors (TLR3, TLR4) was increased, but the expression level of anti-inflammatory molecules (CCL18, CCL22, TREM2, MRC1) was inhibited by a high NaCl concentration [51]. Moreover, human studies have shown that high salt intake increased the number of circulating monocytes and proinflammatory cytokines (IL6, IL23) whereas it reduced the levels of certain anti-inflammatory cytokines (IL10) [52]. The salt-induced increase of IL 23 is of particular importance because this cytokine stimulates the transformation of naïve T cells to Th17 cells [52,53]. In animal models of salt-sensitive hypertension, an association was found between sodium load, renal macrophage infiltration/polarization, and kidney damage [54].

The salt-induced kidney damage has been further documented in mice undergoing subtotal nephrectomy. Salt loading in these animals resulted in macrophage infiltration, epithelial-mesenchymal transformation, and fibrosis. Concomitantly, there was an increase in the mRNA expression of IL6, monocyte chemotactic protein (MCP1), Sgk1, and TonEBP. Chemokine receptor2 (CCR2) deficiency prevented macrophage infiltration of the renal tissue and the development of secondary fibrosis [55]. The salt-induced histopathological damages proved to be independent of blood pressure. Lasting high NaCl intake in normotensive Wistar–Kyoto rats produced renal proximal tubule dilatation, degeneration, and tubulointerstitial fibrosis without blood pressure elevation. These morphological changes were associated with a significant increase of urinary excretion of early biomarkers of renal tubular damage. The increased secretion of vanin-1 appears to be of particular importance because it catalyzes the generation of cysteamine that reduces the activity of antioxidant enzymes and, consequently, contributes to tissue damage by OFR [56].

A recent cross-sectional study of Zambian adult subjects has also shown that a high sodium intake, estimated by measuring the 24 h urinary sodium excretion, was associated with the excretion of 8-isoprostanes, a marker of lipid oxidation, but not with that of 8-OHdG, the marker of oxidative DNA damage [57]. It is of interest that high NaCl has been reported to induce DNA double-strand breaks that are rapidly repaired when NaCl is lowered both in cell culture and in vivo. This reaction appears to be independent of oxidative DNA damage that would be reflected by elevated 8-OHdG production [58].

Further evidence for sodium-dependent oxidative renal tissue injuries has been provided by studies applying antioxidant interventions in salt-sensitive models. For example, hydrogen sulphide (H2S) administration improved renal function and structure, increased antioxidant defense, reduced free oxygen radical generation, and activated factor Nfr2 to promote the expression of target antioxidant enzymes in Dahl rats kept on a high salt diet [59]. Similarly, antioxidant treatment with vitamins C and E decreased the oxygen radical release, reduced renal monocyte/macrophage infiltration, decreased inflammatory cytokine and chemokine expression, and improved renal functions in Dahl rats on a high sodium diet [60].

### 3.2. Myocardium

The direct effects of NaCl causing tissue injury have also been documented in the cardiovascular system. In response to high salt intake, the cardiac macrophage TonEPB–VEGF-C pathway becomes activated with the subsequently increased macrophage infiltration, fibrosis, and lymphangiogenesis in the left ventricular interstitium and perivascular space. As a result, this accelerated remodeling impaired ventricular function, and the hypertension worsened [61]. Further evidence for the prolonged salt-load-induced nonhemodynamic cardiac injury has been provided by Varagic et al.; they reported that excess salt intake significantly impaired diastolic function of both left and right ventricles, increased interstitial and perivascular collagen deposition, and diminished the coronary vasodilatory response to dipyridamole in spontaneous hypertensive ratsbut not in Wistar–Kyoto rats [62]. Human echocardiographic studies confirmed the adverse cardiac effects of high sodium intake. Indeed, daily sodium intake of more than 3.7 g resulted in cardiac hypertrophy, a restructuring of the myocardium, and impaired systolic and diastolic functions [63].

The causal relationship between high-salt-induced OS and myocardial injury has been confirmed by Huang et al. In Dahl rats on a high salt diet, myocardial hypertrophy, decreased α-myosin heavy chain (α-MHC), and increased β-myosin heavy chain (β-MHC) expression were seen. Furthermore, OS in the myocardial tissue was activated as shown by the increased levels of hydroxyl radicals, malondialdehyde, and oxidized glutathione. At the same time, the total antioxidant capacity, carbon monoxide, catalase, glutathione peroxidase, SOD activities, and SOD1/SOD2 protein expression decreased significantly. All these pathological alterations could be inhibited by daily intraperitoneal administration of the powerful antioxidant hydrogen sulfide (H2S) [64].

## 4. Perinatal Pathologies Associated with Sodium Intake and OS

### 4.1. Preeclamptic Pregnancy

It has long been recognized that high salt intake and sodium retention is a risk factor of preeclampsia. In a randomized, cross-over, double-blinded study, high salt intake was found to decrease renin and angiotensin II concentrations in healthy pregnant and nonpregnant women, but it remained unaltered in patients with preeclampsia. Plasma aldosterone was similarly depressed while brain natriuretic peptide increased in all groups. These observations indicated that in preeclampsia, the renal capacity to excrete sodium is impaired, and more sodium is retained when patients are subjected to high sodium intake [65]. Previously, it has also been shown that patients with proteinuric pregnancy-induced hypertension avidly retain sodium but without apparent influences on plasma renin and aldosterone concentrations [66].

In a cross-sectional study of 569 pregnant women from the Odense Child Cohort, urinary sodium and potassium excretion with plasma levels and urinary aldosterone excretion were measured at 29 weeks of gestation to assess their predictive value of feto-placental development and preeclampsia. Salt intake of more than 6 g/kg proved to be an independent predictor of preeclampsia and pregnancy-induced hypertension whereas aldosterone was significantly associated with placental weight and birth weight. Based on these results, it was concluded that high sodium combined with low potassium intake directly increase the risk of preeclampsia. Indirectly, however, suppressing aldosterone production impairs feto-placental development via reducing VEGF and placental growth factor expression [67]. The protective effects of aldosterone on the function of the feto-placental unit have already been established by others [68,69].

In a recent comprehensive review, Scaife and Hohaupt carefully evaluated the association of preeclampsia, salt intake, and aldosterone production and provided evidence that the placenta, similar to the skin interstitium, functions as a salt-sensitive organ and regulates sodium balance [70]. Indeed, the negatively charged GAGa and proteoglycans (syndecan 2, glycopicans 1,3, decorin, perlecan) are abundantly expressed in the placenta of healthy pregnancy and bind and reversibly store excess sodium [71,72]. Accumulated sodium generates hypertonicity that induces macrophage invasion and activation. Activated macrophages release TonEBP and VEGF-C, leading to lymphangiogenesis and eNOS expression. This sodium reservoir can buffer excess sodium and maintain low vascular resistance and utero-placental perfusion. In preeclampsia, the capacity of this system is markedly reduced and cannot meet the actual sodium (and volume) requirements of utero-placental circulation. It is suggested, therefore, that supplemental NaCl should be provided to reestablish normal plasma volume to compensate for the low aldosterone characteristic of preeclampsia. NaCl supplementation had also been claimed to alter immune milieu by preventing a decrease of CD14 macrophages and the unfavourable cytokine balance that may lead to defective placentation [73].

It should be noted that increased levels of tissue sodium have been shown to enhance the polarization of naïve Th cells to Th17 cells, and via their IL 17 production, they may increase the generation of ROS and proinflammatory cytokines that may limit NO bioavailability [74]. These untoward effects of sodium accumulation in the placental tissue are certainly overcome by the benefit of the activation of TonEBP-VEGF-C-eNOS pathways [75].

### 4.2. Prenatal High Sodium Intake and Vascular Programming

The concept of the developmental origin of certain chronic diseases in adulthood was conceived by Barker and coworkers. According to this concept, adverse events at a critical period of development induce long-lasting consequences. Classically, undernourished fetuses adapt to a limited placental nutrient supply to ensure survival but at the expenses of cardiovascular, hormonal, and metabolic reactions that manifest as diseases in adult life. These complex adaptive processes are designated as programming [76,77]. Among early, well-defined pathogenetic factors, increased salt intake emerged as an important player mediating the increased risk of cardiovascular diseases in adults.

In support of this notion, a prenatal high-salt diet in Sprague–Dawly rats has been shown to program blood pressure and heart rate hyperresponsiveness in adult female offspring [78]. Moreover, the adverse effects of excessive sodium intake during pregnancy on the vascular structure of adult offspring have been documented by demonstrating a thicker wall of central arteries, collagen deposition, smooth muscle cell proliferation, and an increased expression of the OS marker, nitrothyrosine [79,80]. The oxidative damage of the vascular wall has been claimed to be accounted for by high levels of circulating cardiotonic steroid marinobufagenin (MBG), which is known to mediate the production of superoxide and peroxynitrite via NADPH oxidase activation [81]. In addition to elevated MBG levels, a reduced expression of eNOS and the NO receptor, soluble guanylate cyclase, with a concomitant increase of circulating ADMA, the competitive inhibitor of NOS, was observed [82]. Elevated asymmetric dimethylarginine (ADMA) levels may be due to redox-sensitive enzyme reactions as its generation by protein methyltransferase (PRMT) is enhanced while its elimination by dimethylarginine dimethylaminohydrolase (DDAH) is inhibited by ROS. In this regard, it should be considered that our group described a markedly elevated plasma ADMA level of premature infants during the first weeks of life and assumed its contribution to the development of cardiovascular diseases in adulthood [83]. Similarly, urinary excretion of endogenous ouabain (EOLS), as a marker of its adrenal secretion, proved to be higher in healthy preterm infants as compared to full-term neonates and remained elevated during the study period of five weeks. When supplemental sodium was given to the preterm infants, urinary EOLS decreased significantly. It was postulated, therefore, that EOLS appears to be regulated by the excessively activated renin-angiotensin stimulation in the nonsupplemented, sodium- and volume-depleted group rather than the volume status of the supplemented groups of preterm neonates [84]. It may also be relevant to note that EOLS proved to be an independent predictor of impaired diurnal blood pressure rhythm and arterial stiffness in adult patients with subclinical organ damage in treated hypertensive patients [85].

### 4.3. Perinatal Adaptation and Oxidative Stress

In a most recent review, Lembo et al. have summarized the most important aspects of our current knowledge on the impact of OS on the perinatal adaptation of preterm neonates [86]. It has been clearly shown that the generation of AFR exceeds the capacity of enzymatic and nonenzymatic antioxidant defense mechanisms during the perinatal period. Therefore, an imbalance develops between the pro- and antioxidant systems, which is designated as OS. Prenatally, reactive oxygen species (ROS) generation is mainly due to the fetal inflammatory response syndrome that comprises infection, hypoxia, and ischemia-reperfusion [87]. In the postnatal care respiratory support, instable circulation, parenteral nutrition, and fluid therapy should be considered as a source of ROS generation. ROS may cause cellular, tissue, and organ damage and induce the so-called free radical-mediated pathologies (bronchopulmonary dysplasia, retinopathy of prematurity, necrotizing enterocolitis, intraventricular haemorrhage, respiratory distress syndrome, and patent ductus arteriosus). Importantly, the association of fluid intake and cardiopulmonary adaptation has been widely studied, and it consistently demonstrated that high, versus restricted, fluid intake negatively influenced these complex processes [88,89,90,91]. To our knowledge, only one study has been performed to address the impact of sodium supplementation, independent of fluid intake, on the cardiopulmonary adaptation. In this randomized, controlled clinical trial, the effects of early (on the second day) and late (when 6% of birth weight was lost) sodium supplementation (4 mmol/kg) were compared on oxygen dependency and body weight in preterm infants with a gestational age of 25–30 weeks. Clinical variables, including fluid and energy intake, were comparable. Preterm infants receiving early sodium supplements needed more respiratory support both on postnatal day 6, and on day 28, but their weight curves and plasma sodium concentrations were similar [92]. The authors did not provide a clear explanation for the more compromised cardiopulmonary adaptation in the early supplemented group, but others suggested that the excess sodium may cause excess volume that is responsible for the respiratory compromise [89].

We suggest that early implementation of a sodium supplement may enhance sodium-induced OS and aggravate the imbalance between pro- and antioxidant defenses.

## 5. Conclusions

It is generally accepted that the volume and tonicity of body fluid compartments is closely associated with the tight control of renal sodium excretion. Inefficient renal sodium excretion may result in the expansion of extracellular space with subsequent volume-dependent, sodium-sensitive hypertension. This concept, however, has been challenged as evidence has been provided for the dissociation of sodium and volume regulation.

The skin interstitium has emerged as a major nonrenal regulator of sodium balance as its negatively charged macromolecules bind osmotically inactive sodium and generate tissue hypertonicity that induce macrophage invasion and activation. Activated macrophages increase the expression of TonEBP and the secretion of VEGF-C that enhance lymphangiogenesis and eNOS expression to drain back the reversibly bound sodium (and volume) into the circulation. Moreover, salt-activated immune cells release cytokines and ROS that may cause progressive tissue damage. A similar sodium buffer/reservoir function has been described in the endothelial glycocalyx and in the placenta. The concept of the nonrenal regulation of sodium balance is greatly appreciated; however, several concerns are to be addressed as follows:

- How and where the chloride, the accompanying anion of sodium, is stored and released.
- Water-free sodium release cannot meet the volume requirements; therefore, bound sodium and bound water should be released simultaneously.
- How the sodium reservoir functions are regulated in the given tissue.
- How we can prevent/attenuate the sodium-induced tissue damage.

With respect to these unanswered questions, we agree with those who propose that excess sodium intake should be avoided at the population level, and at the individual level, sodium intake should be tailored to the actual need determined by meticulous balance studies and a measurement of relevant hormones. Furthermore, to prevent tissue damage, antioxidant, anti-inflammatory, and antifibrotic innovative therapeutic approaches should be implemented.

In summary, nonosmotic tissue sodium storage can be regarded as a two-edged sword. It plays an important role in the control of sodium, in volume homeostasis, and in preventing/attenuating blood pressure elevation but at the expense of inflammatory immune reactions. The latter leads to enhanced ROS generation and to the release of proinflammatory cytokines and profibrotic mediators. These opposing effects of tissue sodium accumulation should be kept in balance to avoid irreversible tissue/organ damage.

## Data Availability

Not applicable.
